# Recognition of acrolein-specific epitopes by B cell receptors triggers an innate immune response

**DOI:** 10.1016/j.jbc.2021.100648

**Published:** 2021-04-09

**Authors:** Ryunosuke Endo, Kazuki Uchiyama, Sei-Young Lim, Masanori Itakura, Takahiro Adachi, Koji Uchida

**Affiliations:** 1 Graduate School of Agricultural and Life Sciences, The University of Tokyo, Tokyo, Japan; 2 Department of Immunology, Medical Research Institute, Tokyo Medical and Dental University, Tokyo, Japan; 3 Japan Agency for Medical Research and Development, CREST, Tokyo, Japan

**Keywords:** lipid peroxidation, post-translational modification (PTM), innate immunity, cell surface receptor, immunogenicity, B cell receptor (BCR), immunoglobulin M (IgM), Abs, antibodies, ACR^−^, acrolein-nonbinding, ACR^+^, acrolein-binding, acrBSA, advanced glycation end products, acrNAK, acrolein-treated *N*^α^-acetyl-L-lysine, BCR, B cell receptor, BSA, bovine serum albumin, CDRH3, heavy-chain complementarity-determining region 3, CFP, cyan fluorescent protein, FDP-lysine, *N*^ε^-3-formyl-3,4-dehydropiperidino-L-lysine, FDP-NAK, *N*^ε^-3-formyl-3,4-dehydropiperidino-*N*^α^-acetyl-L-lysine, IGH, immunoglobulin heavy chain, IGHD, immunoglobulin heavy-chain diversity, IGHJ, immunoglobulin heavy-chain joining, IGHV, immunoglobulin heavy-chain variable, IgM, immunoglobulin M, LDLs, low-density lipoproteins, MACS, magnetic-activated cell sorting, MP-lysine, *N*^ε^-(3-methylpyridinium)-L-lysine, MP-NAK, *N*^ε^-(3-methylpyridinium)-*N*^α^-acetyl-L-lysine, NAb, natural antibody, NAK, *N*^α^-acetyl-L-lysine, PerC, peritoneal cavity, QM mice, quasimonoclonal mice, SPL, spleen, TLRs, toll-like receptors, VH, heavy chain variable region, VL, light chain variable region, YC3.60, yellow cameleon 3.60

## Abstract

Natural antibodies, predominantly immunoglobulin M (IgM), play an important role in the defense against pathogens and in maintaining homeostasis against oxidized molecules known as oxidation-specific epitopes, such as those contained in oxidized low-density lipoproteins. However, owing to the complexity of the oxidized products, very few individual epitopes have been characterized in detail. In the present study, to identify endogenous sources of oxidation-specific epitopes, we stimulated mouse spleen and peritoneal cavity (PerC) cells *in vitro* with bovine serum albumin modified with a variety of lipid peroxidation–related carbonyl compounds and identified the acrolein-modified bovine serum albumin as the most efficient trigger studied for the production of IgM in PerC cells. The acrolein-specific epitopes accelerated the differentiation of B-1a cells, a fetal-derived B cell lineage, to plasma cells. In addition, acrolein-modified bovine serum albumin was specifically bound to B-1a cells, suggesting the presence of an acrolein-specific IgM–B cell receptor (BCR). A hybridoma, RE-G25, producing an acrolein-specific IgM, was established from the PerC cells and was indeed identified as a population of B cells expressing a specific IgM–BCR. In addition, we analyzed the BCR repertoire of acrolein-specific B cells and identified the most frequent IgM heavy chain gene segments of the B cells. These data established the presence of innate B cells expressing the acrolein-specific BCR and suggested that in addition to our understanding of acrolein as a toxic aldehyde, it may play a role as a trigger of the innate immune response.

Innate immunity plays an important role in the defense against pathogens and in maintaining homeostasis. The innate immunity is activated not only by pathogen-associated molecular patterns but also by endogenous danger signals called damage-associated molecular patterns. These disease-associated molecules are known to be recognized by cell surface receptors, such as scavenger receptors and toll-like receptors (TLRs), most of which are expressed in macrophages. They are also accessible for recognition by the soluble pattern recognition receptors, such as natural antibodies (NAbs) (1, 2, 3). The NAbs, predominantly immunoglobulin M (IgM), are mainly produced by B-1 cells, a subtype of B cell lymphocytes that are involved in the humoral immune response. B-1 cells spontaneously secrete NAbs in the absence of external antigen stimulation at tightly regulated levels, which allows the B-1 cells to provide pre-existing, immediate defense to counteract antigens (4, 5, 6). Based on the facts that B-1 cells, particularly IgM-secreting B-1 cells, are already detectable before birth in the fetal liver and at birth in the mouse spleen (SPL) and are among the first B cells to develop, they are considered to be the principal B cells responsible for establishing an NAb repertoire.

Owing to minimal insertion of nontemplate-encoded nucleotides (N-region addition) and little somatic hypermutation (7, 8), the NAbs produced by B-1 cells have a germline-like structure. This is in striking contrast to the high-affinity antibodies (Abs) produced by another subtype of the B lymphocytes, that is, the B-2 cells. The NAbs are often autoreactive and recognize specific molecular patterns with no apparent structural homology (9, 10, 11). Previous studies have shown that the modified self-proteins, such as oxidized low-density lipoproteins (LDLs) and advanced glycation end products, could be the targets of the NAbs (1, 3, 11). Based on these features, the NAbs have been suggested to play an important role in the host defense mechanism against various stresses, as well as in providing immediate protection against pathogens. Furthermore, it has also been reported that the NAbs could be extensively associated with protection against some chronic diseases, such as atherosclerosis (12).

Lipid peroxidation leads to the formation of a wide variety of products with diverse and strong biological activities. Some of the lipid peroxidation products, particularly aldehydes, exhibit reactivity with proteins and produce various intramolecular and intermolecular covalent adducts. Binder and Silverman (13) proposed that such adducts, called oxidation-derived epitopes, produced on self-antigens, are important immunodominant targets for the NAbs. It has been documented that aldehydic species, including short-chain aldehydes and oxidized phospholipid core aldehydes, generated in the *in vitro* oxidized LDL and in the atherosclerotic plaque, could be the source of the oxidation-specific epitopes (1, 13). In addition, malondialdehyde, one of the most abundant lipid peroxidation–derived aldehydes, has been shown to convert proteins into the oxidation-specific epitopes recognized by the NAbs (14). These findings suggest that lipid peroxidation is a major source of oxidation-specific epitopes. However, owing to the complexity of the oxidation-specific epitopes, very few individual innate epitopes have been characterized in detail.

In the present study, among the lipid peroxidation–related carbonyl compounds, we identified acrolein, the most reactive of all α,β-unsaturated aldehydes, as a potential source of the innate epitopes. In addition, we demonstrated that the acrolein-specific epitopes significantly promoted the differentiation of B-1 cells into the IgM-secreting plasma cells and IgM production in the peritoneal cavity (PerC) cells. After identification of the acrolein-lysine adducts as a trigger of the innate immune response, we established the presence of innate B-1 cells that specifically respond to the acrolein-specific epitopes *via* a B cell receptor (BCR)-dependent mechanism. To the best of our knowledge, this is the first study reporting that acrolein has a potential to transform proteins into specific antigens that can activate the innate immune response. This finding may likely reflect the ubiquitous formation of acrolein consequent to various intracellular and extracellular events. Our discovery of the acrolein-specific epitopes as a trigger of the innate immune response also suggests that, besides our common concept of acrolein as a toxic aldehyde, the aldehyde may also be involved in the homeostatic responses by binding to proteins.

## Results

### Stimulation of innate immune response by acrolein-modified proteins

**Figure 1:**
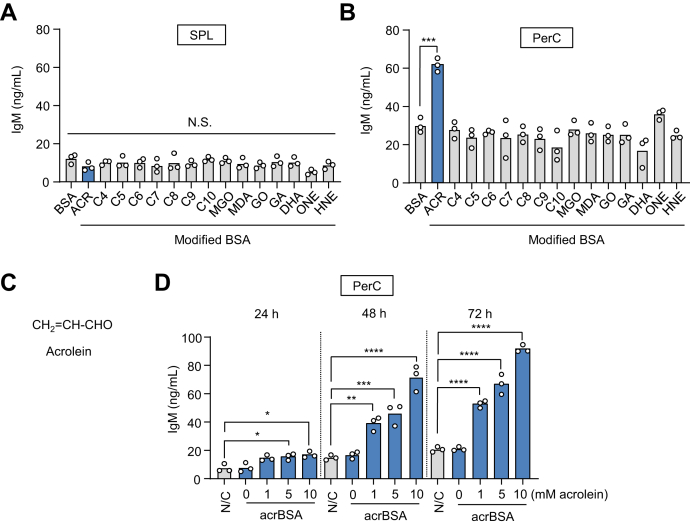
**Stimulation of innate immune response by acrolein-modified proteins.** SPL cells (*A*) and PerC cells (*B*) prepared from native BALB/c mice were treated with control or modified BSAs with lipid peroxidation–related carbonyl compounds, and the production of IgM was examined. Aldehydes used are ACR, acrolein; C4, crotonaldehyde; C5, 2-pentenal; C6, 2-hexenal; C7, 2-heptenal; C8, 2-octenal; C9, 2-nonenal; C10, 2-decenal; MGO, methylglyoxal; MDA, malondialdehyde; GO, glyoxal; GA, glycolaldehyde; DHA, dehydroascorbic acid; ONE, 4-oxo-2-nonenal; HNE, 4-hydroxy-2-nonenal. Differences were analyzed by Dunnett's test; ∗∗∗*p* < 0.001; *versus* BSA. *C*, chemical structure of acrolein. *D*, production of IgM by acrBSA in the PerC cells. The cells were treated with control BSA or acrBSA for the times indicated, and the culture supernatants were tested for the production of IgM. acrBSA was prepared by incubating BSA (1.0 mg/ml) with acrolein (0–10 mM) in PBS for 24 h at 37 °C under atmospheric oxygen. The cells were treated with 100 μg/ml protein samples (control or modified BSAs) for 72 h (*A* and *B*) or 24 to 72 h (*D*). Differences were analyzed by Dunnett's test; ∗*p* < 0.05; ∗∗*p* < 0.01; ∗∗∗*p* < 0.001; ∗∗∗∗*p* < 0.0001; *versus* BSA (*A* and *B*); *versus* N/C within each time condition (*D*). acrBSA, acrolein-modified BSA; BSA, bovine serum albumin; IgM, immunoglobulin M; PerC, peritoneal cavity; N.S., no significance; SPL, spleen.

To identify an unknown source of oxidation-specific epitopes that could trigger the innate immune response, we treated the cells isolated from the SPL and PerC of native BALB/c mice with bovine serum albumin (BSA) modified with lipid peroxidation–related carbonyl compounds and examined the production of IgM. Among a selected set of modified proteins, the Ab production was the most prominently enhanced by the acrolein-modified BSA (acrBSA) in the PerC cells, whereas no significant response to the modified proteins was observed in the SPL cells (Fig. S1 and Fig. 1, *A*–*C*). The IgM production was dependent on the concentration of acrolein used for the modification of the protein and on the time spent in culture (Fig. 1*D*). These data and the fact that acrolein could be ubiquitously generated in biological systems (15) suggest the existence of an association between the formation of acrolein as a source of the oxidation-specific epitopes and the innate immune response.

### Involvement of B-1a cells in the innate immune response triggered by acrolein-modified proteins

**Figure 2:**
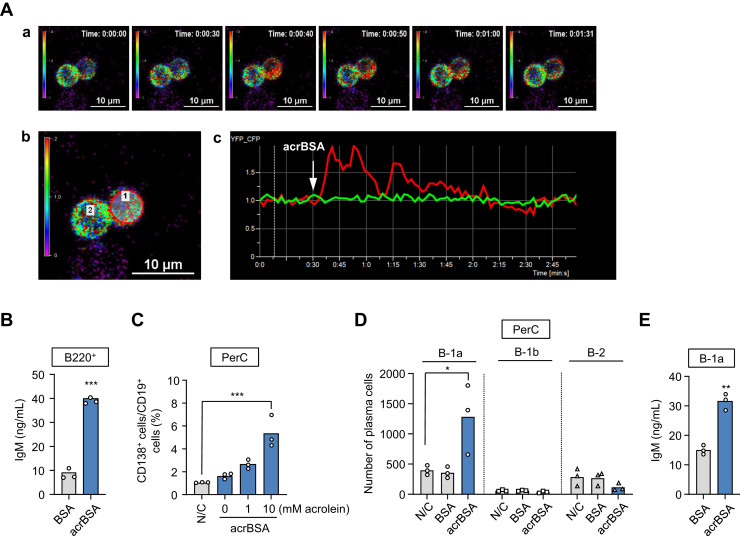
**Involvement of B-1a cells in the innate immune response triggered by acrolein-modified proteins.***A*, acrBSA-induced Ca^2+^ flux in PerC B cells *in vitro*. *a*, representative Ca^2+^ signaling images in PerC B cells from CD19-Cre/YC3.60^fl/fl^ mice. Ratiometric images (YFP/CFP excitation at 458 nm) are shown. acrBSA in PBS (final concentration: 10 μg/ml) was added to the cell culture at the indicated time point. A rainbow parameter indicates the relative Ca^2+^ concentrations. *b*, the indicated cells were measured fluorescence intensities of the YFP/CFP ratio upon excitation at 458 nm. *c*, time course for fluorescence intensities of the YFP/CFP ratio upon excitation at 458 nm in the cells. *B*, production of IgM by acrBSA in the PerC B cells. B cell subpopulations (B220^+^) were isolated from the PerC of BALB/c mice by MACS MicroBeads UltraPure, stimulated with BSA or acrBSA, and the culture supernatants were tested for the production of IgM. Differences were analyzed by a paired Student's *t* test; ∗∗∗*p* < 0.001. *C*, differentiation of B cells into the IgM-secreting plasma cells by acrBSA. *D*, identification of B cell subsets involved in the production of IgM by acrBSA. A total of the 10,000 events were counted and analyzed. *E*, production of IgM by acrBSA in the B-1a cells. The PerC B-1a cells were stimulated with BSA or acrBSA, and the culture supernatants were tested for the production of IgM. In *panels B–E*, the cells were treated with 100 μg/ml protein samples (BSA or acrBSA) for 48 h (*B*, *C*, and *E*) or 72 h (*D*). Differences were analyzed by the paired Student's *t* test (*B* and *E*) or by Dunnett's test (*C* and *D*); ∗*p* < 0.05; ∗∗*p* < 0.01; ∗∗∗*p* < 0.001; *versus* N/C (*C*); *versus* N/C within each B cell subset (*D*). acrBSA, acrolein-modified BSA; IgM, immunoglobulin M; MACS, magnetic-activated cell sorting; N/C, negative control; PerC, peritoneal cavity; YC3.60, yellow cameleon 3.60.

To demonstrate that the enhanced IgM production by acrBSA was due to its action with the PerC B cells, we assessed the B cell response stimulated by acrBSA using intravital imaging of B cell–specific yellow cameleon 3.60 (YC3.60) transgenic mice (16). The time-lapse observation revealed that acrBSA transiently induced Ca^2+^ flux in the single PerC B cell (Fig. 2*A*), indicating that acrBSA could act on the B cells as an external stimulus.

To directly demonstrate that IgM Abs are derived from PerC B cells, we isolated the B cell subpopulation (B220^+^) from the PerC of BALB/c mice by MACS MicroBeads UltraPure, stimulated them with BSA or acrBSA, and tested the culture supernatants for the production of IgM. Figure 2*B* shows that acrBSA caused a significant IgM production in the B cells. In addition, acrBSA promoted the differentiation of B cells into the IgM-secreting plasma cells (Fig. 2*C*). To identify the B cell subsets involved in the production of IgM by the acrolein-modified proteins, the PerC cells treated with the native BSA or acrBSA were sorted into the subsets, B-1a, B-1b, and B-2 cells, and the changes in the number of plasma cells were measured. The result showed that acrBSA caused a significant increase in the number of B-1a-derived plasma cells, whereas no significant change in the number of plasma cells that originated from B-1b and B-2 cells was observed (Figs. 2*D* and S2). Moreover, acrBSA, but not the native BSA, accelerated the production of IgM in the isolated PerC B-1a cells (Fig. 2*E*). These data indicated that the acrolein-specific epitopes account for the IgM response induced by the B-1a cells.

### Identification of acrolein-specific innate epitopes

**Figure 3:**
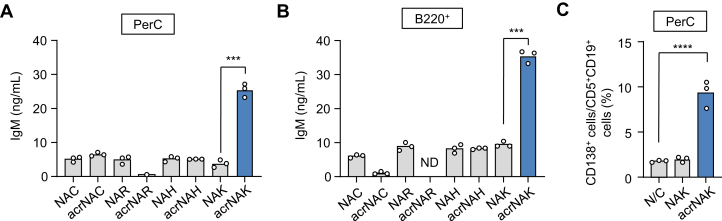
**Identification of an amino acid involved in the formation of acrolein-specific innate epitopes.***A*, production of IgM by acrolein-treated amino acids in the PerC cells. *B*, production of IgM by acrolein-treated amino acids in the PerC B cells. Differences were analyzed by a paired Student's *t* test; ∗∗∗*p* < 0.001. *C*, enhanced differentiation of B cells into the plasma cells by acrolein-treated NAK in the PerC cells. In *panels A–C*, the reaction mixture containing 10 mM *N*^*α*^-acetylated amino acids and 1 mM acrolein in PBS was incubated for 24 h at 37 °C. The cells were treated with amino acids (control or acrolein-treated amino acids) for 48 h. Differences were analyzed by the paired Student's *t* test (*A* and *B*) or by Dunnett's test (*C*); ∗∗∗*p* < 0.001; ∗∗∗∗*p* < 0.0001; compared within each amino acid (*A* and *B*); *versus* N/C (*C*). IgM, immunoglobulin M; NAK, *N*^α^-acetyl-L-lysine; PerC, peritoneal cavity.

It has been shown that, upon reaction with proteins, acrolein reacts with the nucleophilic amino acids, such as cysteine, histidine, and lysine residues (15). To identify the acrolein-specific epitopes responsible for the innate immune response, the *N*^α^-acetyl derivatives of the amino acids that had been incubated with acrolein were tested for their ability to trigger IgM production in the PerC cells *in vitro*. As shown in Figure 3*A*, among the *N*^α^-acetylated amino acids tested, only the acrolein-treated *N*^α^-acetyl-L-lysine (acrNAK) showed the greatest activity in enhancing the production of IgM. Similarly, the acrNAK enhanced the production of IgM in the B cell subpopulation isolated from the PerC (Fig. 3*B*). In addition, when the PerC cells were treated with the acrNAK, an enhanced differentiation of the B-1a cells into the plasma cells was observed (Fig. 3*C*). These data strongly suggest that the acrolein-lysine adduct(s) could induce the differentiation of B-1a cells as an innate antigen.

**Figure 4:**
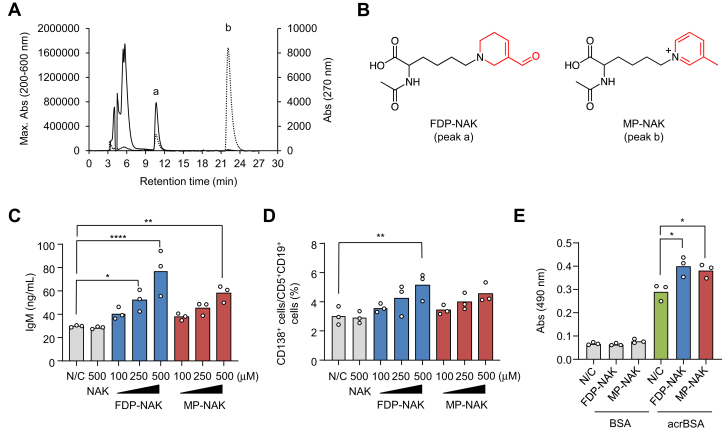
**Identification of acrolein-specific innate epitopes**. *A*, reverse-phase HPLC analysis of acrolein-treated NAK. The reaction was performed by incubating 10 mM NAK with 1 mM acrolein in PBS (pH 7.4) for 24 h at 37 °C. The reaction was monitored by the absorption maximum at 200 to 600 nm (*solid line*) and at 270 nm (*dotted line*). *B*, chemical structure of *N*^ε^-3-formyl-3,4-dehydropiperidino-*N*^α^-acetyl-L-lysine (FDP-NAK) (peak a) and *N*^ε^-(3-methylpyridinium)-*N*^α^-acetyl-L-lysine (MP-NAK) (peak b). *C*, dose-dependent production of IgM by FDP-NAK and MP-NAK in the PerC cells. *D*, dose-dependent differentiation of B-1a cells into the plasma cells by FDP-NAK and MP-NAK. *E*, antigen-binding activity of the IgM Abs produced by the cells that had been treated with the acrolein-specific adducts (FDP-lysine and MP-lysine). The PerC cells were treated with 0.5 mM NAK or 0.1 to 0.5 mM acrolein-lysine adducts (FDP-NAK and MP-NAK) for 72 h at 37 °C, and the culture supernatants were tested for the cross-reactivity with the control BSA or acrBSA. Differences were analyzed by Dunnett's test; ∗*p* < 0.05; ∗∗*p* < 0.01; ∗∗∗∗*p* < 0.0001; *versus* N/C (*C* and *D*); *versus* N/C within each IgM response to the same antigen (*E*). Abs, antibodies; acrBSA, acrolein-modified BSA; BSA, bovine serum albumin; IgM, immunoglobulin M; NAK, *N*^α^-acetyl-L-lysine.

To identify the active component generated upon the incubation of *N*^α^-acetyl-L-lysine (NAK) with acrolein, NAK was incubated with an equimolar concentration of acrolein in 0.1 M phosphate buffer (pH 7.4) for 24 h, and the reaction mixtures were analyzed by reverse-phase HPLC monitored with the absorption maximum at 200 to 600 nm and at 270 nm. As shown in Figure 4*A*, two major products, a and b, were detected. Based on the NMR and MS analyses, the products, a and b, were identified as *N*^ε^-3-formyl-3,4-dehydropiperidino-*N*^α^-acetyl-L-lysine (FDP-NAK) and *N*^ε^-(3-methylpyridinium)-*N*^α^-acetyl-L-lysine (MP-NAK), respectively (Fig. 4*B*), which had been reported to be formed upon the reaction of NAK with acrolein under physiological conditions (17, 18, 19). When both FDP-NAK and MP-NAK were subjected to the IgM production in the peritoneal cells *in vitro*, they significantly enhanced the production of IgM in dose-dependent manners (Fig. 4*C*). Similarly, the differentiation of B-1a cells into the Ab-secreting plasma cells was moderately enhanced by the adducts (Fig. 4*D*). However, they were barely effective for the differentiation of the B-1b and B-2 cells (data not shown). To further determine the specificity of the Abs produced in response to these adducts by the PerC cells, we incubated the PerC cells with FDP-NAK or MP-NAK *in vitro* and examined the antigen (acrBSA)-binding activity of the IgM Abs produced by the cells. The result demonstrated a significant increase in the levels of IgM binding to the antigen (Fig. 4*E*). Thus, both the Nε-3-formyl-3,4-dehydropiperidino-L-lysine (FDP-lysine) and Nε-(3-methylpyridinium)-L-lysine (MP-lysine) adducts may, at least in part, contribute to the innate response triggered by the acrolein-modified proteins. However, the fact that the IgM production and B cell differentiation required semimicromolar concentrations (>100 μM) of both adducts suggests the involvement of acrolein-specific antigens other than the FDP-lysine and MP-lysine adducts.

### A BCR-dependent IgM response to acrolein-specific epitopes

**Figure 5:**
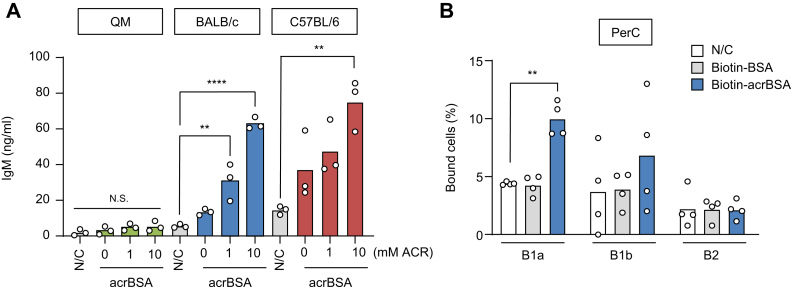
**BCR-dependent IgM response to acrolein-specific epitopes.***A*, PerC cells prepared from QM, BALB/c, and C57BL/6 mice were treated with the control BSA or acrBSA for 48 h at 37 °C, and the culture supernatants were tested for the production of IgM. acrBSA was prepared by incubating BSA (1.0 mg/ml) with acrolein (0–10 mM) in PBS for 24 h at 37 °C under atmospheric oxygen. *B*, binding of biotin–BSA or biotin–acrBSA to the B-1a cells. The cells bound to the antigens are depicted as a percentage of the total B-1a cells. Differences were analyzed by Dunnett's test; ∗∗*p* < 0.01; ∗∗∗∗*p* < 0.0001; *versus* N/C within each group (*A* and *B*). acrBSA, acrolein-modified BSA; BCR, B cell receptor; BSA, bovine serum albumin; IgM, immunoglobulin M; PerC, peritoneal cavity; QM, quasimonoclonal.

To determine if the immune response to the acrolein-specific epitopes are BCR dependent, we used the PerC cells from the quasimonoclonal (QM) mice that provide a model to analyze the B cell selection because of the specificity of BCR to (4-hydroxy-3-nitrophenyl)acetyl. As shown in Figure 5*A*, in contrast to the significantly higher responses of the peritoneal cells from the BALB/c and C57BL/6 mice to acrBSA, the cells from the QM mice showed a very weak response to the antigen, suggesting that the IgM response accelerated by the acrolein-specific epitopes might account for the IgM–BCR expressed on the B cells. To further address the presence of a putative acrolein-specific BCR on the B-1a cells, we carried out a binding assay using biotinylated acrolein-modified proteins (Fig. S3) and observed that the acrolein-modified protein exhibited selective binding to the B-1a cells (Fig. 5*B*).

### Identification of an acrolein-specific IgM–BCR

**Figure 6:**
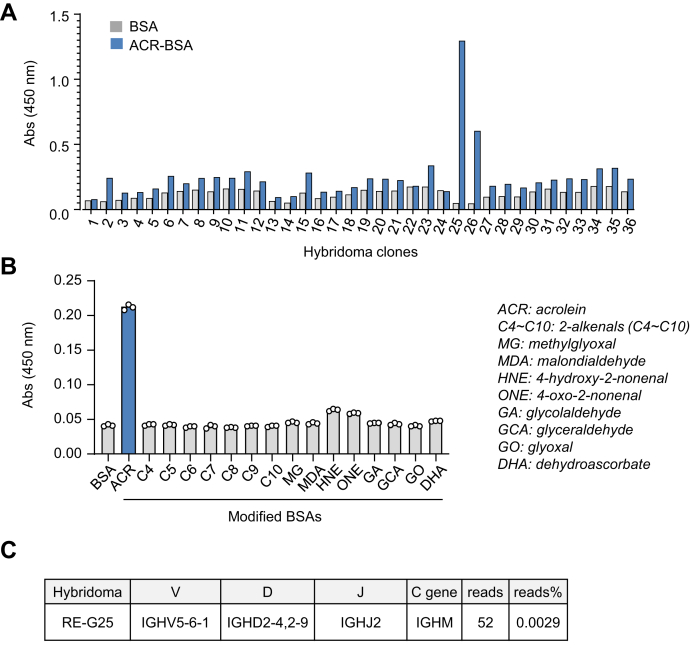
**Identification of an acrolein-specific IgM–BCR.***A*, the hybridoma clones showing a specificity toward acrBSA. The PerC cells were fused with P3U1 mouse myeloma cells and, after screening based on the specific binding to acrBSA, 36 hybridoma clones were obtained. *B*, specificity of IgM mAb produced by hybridoma clone RE-G25 (clone No. 25). *C*, VDJ gene usage of VH for RE-G25. acrBSA, acrolein-modified BSA; BCR, B cell receptor; IgM, immunoglobulin M.

To establish the presence of an acrolein-specific BCR, the PerC cells fused with P3U1 mouse myeloma cells and 36 hybridoma clones, showing specificity toward acrBSA, were obtained. After a screening based on the specific binding to the corresponding antigen, one acrolein-specific hybridoma clone RE-G25 (clone No. 25) was established (Fig. 6*A*). The IgM mAb produced by RE-G25 showed the highest sensitivity and specificity to the acrolein-modified proteins (Fig. 6*B*).

Ab variable regions are composed of a heavy (heavy chain variable region [VH]) and a light (Light chain variable region [VL]) chain. During the early development of each B cell, a functional heavy chain variable domain is created by the essentially random recombination of three germline genes, namely immunoglobulin heavy-chain variable (IGHV), immunoglobulin heavy-chain diversity (IGHD), and immunoglobulin heavy-chain joining (IGHJ) genes, that are each selected from a separate set. Similarly, a functional light-chain variable domain is created by V-J recombination. The process is known as V(D)J recombination, which is a critical mechanism for the diversity and specificity of the Abs. To characterize the genetic origin of the acrolein-specific IgM mAbs, VH and VL genes were amplified, cloned, and sequenced. The sequences were compared with germline sequences in the ImMunoGeneTics information system/V-QUEST database to identify the most homologous germline genes and to detect somatic mutations. The V-D-J gene usage of the VH and VL for the RE-G25 hybridoma displayed a 100% match to the germline gene sequences (Figs. S4 and S5). In addition, the hybridoma was identified as a population of B cells expressing IGHV5-6-1–encoded IgM–BCR, composed of the IGHD2-4 and IGHD2-9 gene, and IGHJ2 gene (Fig. 6*C*). Thus, the IgM mAb cloned from the PerC cells might represent an expansion of the population of NAb ubiquitously expressed in healthy individuals.

### Comparison of V gene usage by acrolein-binding and acrolein-nonbinding B cell populations

**Figure 7:**
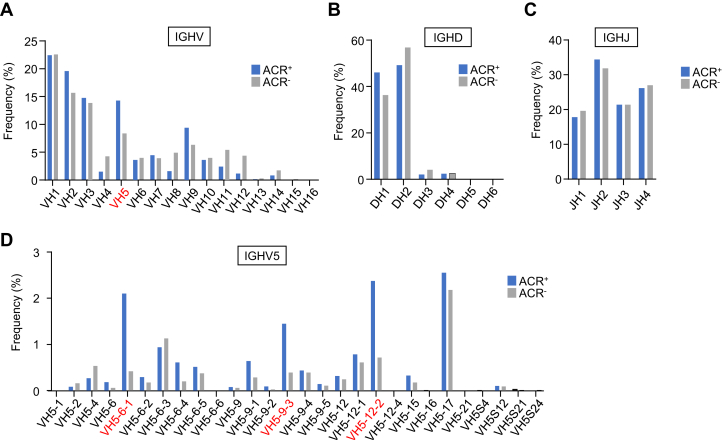
**Comparison of the usage of IGHV, IGHD, and IGHJ genes of IgM–BCRs in ACR**^**+**^**and ACR**^**−**^**PerC B cell populations.** The PerC B cells isolated from BALB/c mice were fractionated into the ACR^+^ and ACR^−^ B cell populations by flow cytometry. The mean percentage usages of IGHV, IGHD, and IGHJ are shown. The *bars* indicate the mean percentage usage of ACR^+^ (*blue*) and ACR^−^ (*gray*) B cells. *A*, IGHV gene usage. *B*, IGHD gene usage. *C*, IGHJ gene usage. *D*, usages of the IGHV5 gene segments. ACR^−^, acrolein-nonbinding; ACR^+^, acrolein-binding; BCR, B cell receptor; IGHD, immunoglobulin heavy-chain diversity; IGHJ, immunoglobulin heavy-chain joining; IGHV, immunoglobulin heavy-chain variable; IgM, immunoglobulin M.

Next-generation sequencing technologies, which can generate large amounts of sequence information, has dramatically improved, and Ab repertoire analysis has become indispensable for the identification of antigen-specific Ab genes. To quantify the frequency of the IgM heavy-chain gene sequences, we sequenced the immunoglobulin heavy-chain genes of fluorescence-activated cell sorting–sorted acrolein-binding (ACR^+^) and acrolein-nonbinding (ACR^−^) B cell populations isolated from BALB/c mice (Fig. S6) and compared the usage of the gene segments (IGHV, IGHD, and IGHJ). The result showed that the ACR^+^ B cells were enriched for the IGHV5 clonotypes in the IGHV gene usage relative to the ACR^−^ B cells (Fig. 7*A*), whereas no differences in the IGHD and IGHJ gene usages between the two populations (Fig. 7, *B* and *C*). Further analysis of the ACR^+^ and ACR^−^ B cell repertoires focused on individual gene segments, showing preferential usages of the IGHV5 gene segments, particularly IGHV5-6-1, IGHV5-9-3, and IGHV5-12-2, in the ACR^+^ B cells than in the ACR^−^ B cells (Fig. 7*D*). Predominant usages of gene segments, such as IGHV1-14, IGHV1S137, IGHV3-8, IGHV39-1, IGHV9-3-1, and IGHV9-4, were also observed in the ACR^+^ B cells relative to the ACR^−^ B cells (data not shown).

### Heavy-chain complementarity-determining region 3 characteristics of IgM–BCRs using the IGHV5-6-1, IGHV5-9-3, and IGHV5-12-2 segments

**Figure 8:**
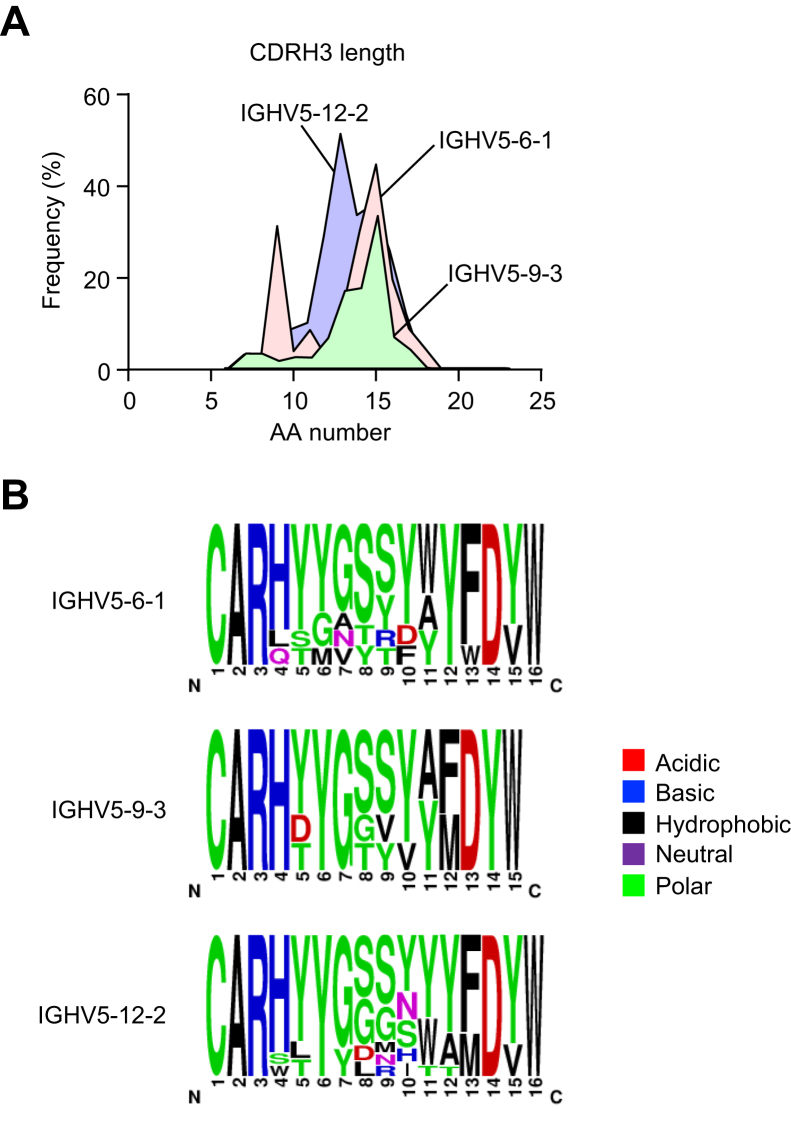
**CDRH3 characteristics of IgM–BCRs using the IGHV5-6-1, IGHV5-9-3, and IGHV5-12-2 segments**. *A*, CDRH3 length distribution of pooled IGH gene sequences of identified IGHV families of the ACR^+^ B cells. *B*, CDRH3 consensus logos of the mode CDRH3 length for pooled IGHV5-6-1, IGHV5-9-3, and IGHV5-12-2 IGH gene sequences. ACR^+^, acrolein-binding; BCR, B cell receptor; CDRH3, heavy-chain complementarity-determining region 3; IGH, immunoglobulin heavy chain; IgM, immunoglobulin M.

The heavy-chain complementarity-determining region 3 (CDRH3) of immunoglobulin molecules is critical for specificity and affinity. We examined the structures of the CDRH3 region of the IgM–BCRs expressed on B cell clones carrying the IGHV5-6-1, IGHV5-9-3, and IGHV5-12-2 segments. Table 1 shows the deduced amino acid sequences of CDRH3 of the IgM–BCRs using these three segments in the ACR^+^ B cells. They mainly use IGHD1-1 followed by IGHD2-3. We further examined the CDRH3 length and found that the average CDRH3 length did not significantly differ among the three gene segments (Fig. 8*A*). It is worth noting that the CDRH3 sequence (CARHDYDYW) of IgM–BCR expressed on the IGHV5-6-1–carrying acrolein-specific hybridoma RE-G25 was considerably shorter than those of the IgM–BCRs listed in Table 1. Pockets of polar amino acids flanked by hydrophobic residues were common to the CDRH3 of the IGHV5-6-1–, IGHV5-9-3–, and IGHV5-12-2–encoded receptors (Fig. 8*B*). They contained multiple polar tyrosine residues, which might play a role in making favorable contacts with the acrolein-specific epitopes. In contrast, they carried less charged amino acids, except for the highly conserved arginine and aspartic acid.

**Table 1:** Deduced amino acid sequences of CDR3 of IgM–BCRs using the IGHV5-6-1, IGHV5-9-3, and IGHV5-12-2 segments

IGHV	IGHD	IGHJ	IGHC	CDR3 AA sequence	Number of reads
IGHV5-6-1	X	IGHJ3	IGHM	CARLTGAYW	884
IGHV5-6-1	IGHD1-1	IGHJ2	IGHM	CARHYYGSSYYFDYW	379
IGHV5-9-3	IGHD1-1	IGHJ2	IGHM	CARHYYGSSYYFDYW	378
IGHV5-12-2	IGHD1-1	IGHJ2	IGHM	CARHYYGSSYYFDYW	276
IGHV5-6-1	IGHD1-1	IGHJ3	IGHM	CARHYYGSSYAYW	223
IGHV5-12-2	IGHD2-9, 2-4	IGHJ4	IGHM	CARSTMITTMDYW	217
IGHV5-12-2	X	IGHJ4	IGHM	CARHGNYAMDYW	191
IGHV5-6-1	IGHD2-3	IGHJ2	IGHM	CARHDGYFDYW	149
IGHV5-12-2	IGHD2-3	IGHJ2	IGHM	CARHDGYYYFDYW	121
IGHV5-12-2	IGHD2-8, 2-11, 2-10	IGHJ1	IGHM	CARHGNYWYFDVW	95
IGHV5-9-3	IGHD1-1	IGHJ4	IGHM	CARHTTVVAMDYW	91
IGHV5-12-2	IGHD1-1	IGHJ1	IGHM	CARHYYGSSYWYFDVW	88
IGHV5-6-1	X	IGHJ2	IGHM	CARHDYW	86
IGHV5-12-2	IGHD1-1	IGHJ2	IGHM	CARHYYYGSSYYFDYW	86
IGHV5-12-2	IGHD2-3	IGHJ4	IGHM	CARWLLRYYAMDYW	85
IGHV5-12-2	IGHD2-8, 2-11, 2-10	IGHJ4	IGHM	CARHGNYAMDYW	82
IGHV5-6-1	IGHD2-3	IGHJ4	IGHM	CARHYDGYYGAMDYW	81
IGHV5-12-2	IGHD1-1	IGHJ2	IGHM	CARHYGSSYYFDYW	79
IGHV5-12-2	IGHD1-1	IGHJ1	IGHM	CARHYYGSSWYFDVW	76
IGHV5-12-2	X	IGHJ2	IGHM	CARHWYFDYW	58
IGHV5-12-2	IGHD1-1	IGHJ2	IGHM	CARHYYGSSYFDYW	56
IGHV5-6-1	IGHD2-9, 2-4	IGHJ2	IGHM	CARHDYDYW	52
IGHV5-6-1	IGHD2-11, 2-10	IGHJ2	IGHM	CARHSMVTTYYFDYW	50

BCRs, B cell receptors; IGHC, immunoglobulin heavy chain constant region; IGHD, immunoglobulin heavy-chain diversity; IGHJ, immunoglobulin heavy-chain joining; IGHM, immunoglobulin heavy constant mu; IGHV, immunoglobulin heavy-chain variable; IgM, immunoglobulin M.

**Figure 9:**
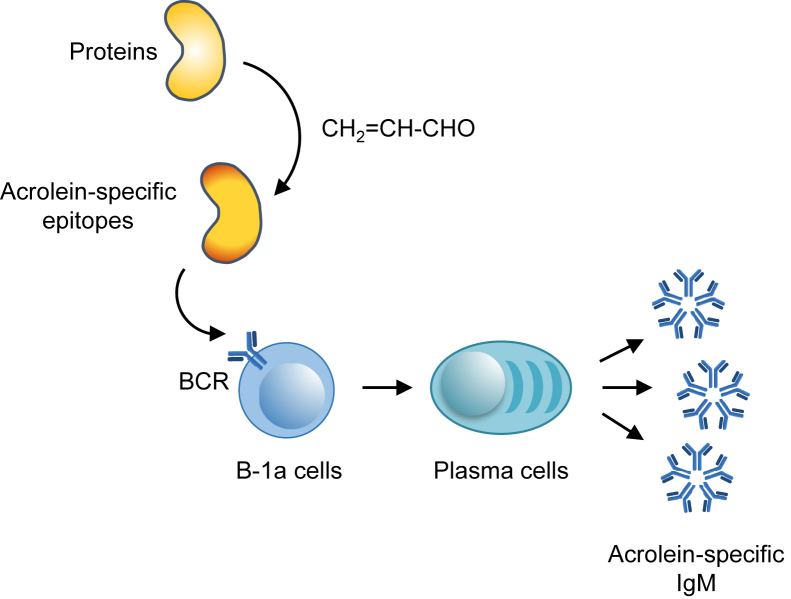
**Schematic representation of the mechanism by which acrolein-specific epitopes accelerate the conversion of B-1a cells to the IgM-secreting plasma cells *via* binding to a specific IgM–BCR.** BCR, B cell receptor; IgM, immunoglobulin M.

## Discussion

Several endogenous substrates are converted to acrolein. It has been reported that an amino acid, threonine, and polyamines are enzymatically converted to acrolein by myeloperoxidase and polyamine oxidase, respectively (20, 21). In addition, the peroxidation of polyunsaturated fatty acids is known to be an efficient source of acrolein (17, 18, 22). Acrolein is also produced during the biotransformation of allyl compounds and the widely used anticancer drug, cyclophosphamide. Besides these mechanisms associated with biological phenomena, acrolein is known to be a ubiquitous molecule in the environment. The exogenous sources of this aldehyde include the combustion of fossil fuels, including engine exhausts, wood, and tobacco, and the heating of cooking oils. Acrolein, among the 2-alkenals, is by far the strongest electrophile and gives rise to the covalent modification of proteins by reacting with the side chains of cysteine, histidine, and lysine residues. In particular, the reaction with lysine residues is unique, generating the most stable products, FDP- and MP-lysine adducts (17, 19). The present study revealed that the acrolein-modified proteins could accelerate the conversion of the B cells to the IgM-secreting plasma cells (Fig. 9). In addition, B-1a cells, among the B cell subsets, were found to be responsible for the IgM response induced by the acrolein-modified proteins. After the identification of the acrolein-lysine adducts, such as FDP-lysine and MP-lysine, as a trigger of the innate immune response, we established the presence of innate B-1a cells that specifically respond to the acrolein-specific epitopes *via* a BCR-dependent mechanism. The data established for the first time that acrolein has a potential to transform proteins into specific antigens that can activate the innate immune response.

B-1 cells, a predominant B cell subset in PerC, play an important role in the innate immunity. The cells produce IgM NAbs, which not only provide a front-line defense against many bacterial and viral pathogens but serve to maintain tissue homeostasis through the recognition of antigenic epitopes (2, 3). Strikingly, up to 30% of the B-1 cell–derived IgM NAbs has been reported to bind to oxidation-specific epitopes (1). However, few studies have addressed the presence of B cells that specifically recognize a particular oxidation-specific epitope. This may be partly because of difficulties in identifying such rare cells. In the present study, we provided evidence that the B-1a subset in the PerC could positively respond to the acrolein-specific epitopes. This finding indicated that the acrolein-specific epitopes could function as a receptor agonist stimulating the production of IgM by the B-1a cells. We indeed observed that the innate immune response by the acrolein-specific epitopes was completely abolished in the 4-hydroxy-3-nitrophenylacetyl–specific BCR knock-in QM mouse model (Fig. 5*B*). In addition, both TLR2 and TLR4 agonists failed to stimulate the production of IgM in the PerC culture (data not shown). These data suggest that the activation of the B-1a cells followed by the production of IgM in response to the acrolein-specific epitopes might be driven by a BCR-dependent mechanism.

B cells express the clonotype-specific surface IgM, constituting the BCR. Binding of the specific antigen to the IgM–BCR triggers a signaling cascade that leads, in concert with other signals, to cell activation, proliferation, and generation of memory B cells and plasma cells. To gain insight into the acrolein-specific IgM–BCRs, we generated hybridomas from the PerC cells of normal BALB/c mice and found that one hybridoma RE-G25 generated an IgM mAb, showing specificity toward the acrolein-modified proteins. In addition, we sequenced the IGH gene of the hybridoma and found that the cell, displaying complete germline gene usage of the VH and VL rearrangements, was a population of B cells expressing the IGVH5-6-1–encoded BCR (Fig. 6). Although definitive information is not available, it is notable that the light-chain variable region of IgM mAb RE-G25 shared a 99% identity with that of an Ab reported in a study about human natural autoantibodies reacting with histone (23). Furthermore, to quantify the frequency of the IgM heavy-chain gene sequences expressing individual VH, DH, and JH genes, we sequenced the IGH genes of the ACR^+^ and ACR^−^ B cell populations isolated from BALB/c mice and found that the ACR^+^ B cell populations were enriched for IGHV5 clonotypes relative to the ACR^−^ B cells (Fig. 7). Distinct differences in the usage of individual VH segments, particularly preferential usage of IGHV5 gene segments, such as IGHV5-6-1, IGHV5-9-3, and IGHV5-12-2, were observed in the ACR^+^ B cells as compared with the ACR^−^ B cells. This finding and the usage of IGVH5-6-1 in the acrolein-specific hybridoma RE-G25 suggest that acrolein-specific IgM–BCRs may be preferentially encoded by the IGVH5 family genes.

The complementarity-determining regions are generally responsible for the antigen recognition of immunoglobulins. CDRH3 among the complementarity-determining regions is particularly critical for specificity and affinity, and therefore, alterations in the CDRH3 properties could reflect the selection for certain antigens. The deduced amino acid sequences of CDR3 of the IgM–BCRs demonstrated the preferential usage of the rearranged IGHV5-6-1 gene by the circulating IgM-expressing ACR^+^ B cells (Table 1). This is consistent with the usage of the IGVH5-6-1 gene by the acrolein-specific hybridoma RE-G25, suggesting that these restricted repertoires may enable the cells to respond to acrolein-specific epitopes. To gain more insight into the preferential usage of the IGHV5 family genes, we examined the structures of the CDRH3 region of the IgM–BCRs expressed on B cell clones carrying the IGHV5-6-1, IGHV5-9-3, and IGHV5-12-2 segments. The CDRH3 sequences demonstrated (i) abundant polar amino acids flanked by hydrophobic amino acid residues and (ii) the highly conserved charged amino acids, such as arginine and aspartic acid, that could contribute specificity to molecular recognition by establishing precise electrostatic and hydrogen-bonding interactions (Fig. 8). The abundance of tyrosine is particularly notable. Tyrosine, among the polar amino acids, is known to play a role in eliminating large volumes of space and forming hydrogen bonds, hydrophobic interactions, and electrostatic interactions with positively charged groups, therefore mediating most of the contacts necessary for high-affinity antigen recognition (24, 25, 26). These chemical properties may partly contribute to both the specificity and affinity to the positively charged acrolein-specific epitopes, such as FDP-lysine and MP-lysine.

FDP-lysine and MP-lysine are two of the major acrolein-specific adducts generated during the lipid peroxidation modification of proteins (17, 18, 22). In our previous study, to verify the presence of these adducts *in vivo*, we obtained a murine monoclonal Ab 5F6 that clearly distinguished the acrolein-modified protein from the native protein and found that the Ab could specifically recognize both FDP-lysine and MP-lysine (19). Using this Ab, we revealed that the acrolein-specific epitopes could be formed in the atherosclerotic lesions, in which the intense positivity is primarily associated with macrophage-derived foam cells (18, 27). We have also revealed that the atherosclerosis-prone apolipoprotein E–deficient mice display a significant elevation of the IgM responses to the acrolein-specific epitopes (Lim, S.-Y., Itakura, M., and Uchida, K., unpublished observation). These findings may likely reflect the ubiquitous formation of acrolein consequent to various intracellular events, including lipid peroxidation. Given the fact that the peroxidation of LDL is associated with the production of acrolein and its adducts in the apolipoprotein molecule (17, 18), the acrolein–lysine adducts may represent one of the oxidation-specific epitopes recognized by the innate immune system (1, 3).

In conclusion, the results of this study raised the possibility that acrolein could be a potential source of innate epitopes. In addition, the presence of innate B cells, expressing the acrolein-specific BCR, suggest that the aldehyde-specific epitopes may play a role as a trigger of the innate immune response. However, it remains unclear if acrolein and/or acrolein-specific antigens directly influence the clonal selection of immature B cells or drive the expansion of rare clonotypes present in the peripheral mature naive B cell pool. Although the mechanisms leading to the conversion of B-1a cells to plasma cells and production of acrolein-specific IgM Abs are poorly understood, these findings may likely reflect the ubiquitous formation of acrolein consequent to various intracellular and extracellular events. Thus, our discovery of acrolein as a source of innate epitopes provides a key link connecting oxidative events and innate immunity and suggests that, besides our common concept of acrolein as a toxic aldehyde, the aldehyde may also play a role as a crucial signal for cell survival (also called a tonic signal) mediating the homeostatic responses *via* binding to proteins (28). Future work is therefore needed to understand the role of the acrolein-specific BCR in mediating the biological effects of acrolein-modified proteins and to determine whether acrolein could contribute to the innate immunity *in vivo*.

## Experimental procedures

### Materials

BSA was obtained from Wako Pure Chemical Industries, Ltd. Acrolein was obtained from Tokyo Kasei. 4-Hydroxy-2-nonenal and 4-oxo-2-nonenal were prepared as previously reported (29, 30). Biotinylated BSA was prepared by incubating 5 mg/ml BSA with a 10-fold molar biotin-PE-maleimide (Dojindo Laboratories) in PBS (−) at 25 °C for 16 h. After incubation, the aliquots were dialyzed against PBS. All other reagents used in the study were of analytical grade and obtained from commercial sources.

### Mice

BALB/c and C57BL/6 mice were purchased from CLEA Japan Inc and maintained according to the guidelines set forth by the Animal Experiment Committee in the Graduate School of Agricultural and Life Sciences, The University of Tokyo. QM mice were provided by Prof M. Wabl (University of California, San Francisco) (31). The YC3.60 mice were established as described previously (16). The YC3.60 mice were intercrossed with CD19-Cre transgenic mice (CD19-Cre/YC3.60) to induce specific transgenic expressions in the B cells and maintained under specific pathogen-free conditions according to the guidelines set forth by the animal committee of the Tokyo Medical and Dental University. The YC3.60^fl/fl^ mouse line was intercrossed with the CD19-Cre knock-in mouse line (32). The resulting CD19-Cre/YC3.60^fl/fl^ mouse line, which specifically expresses YC3.60 in B cells, was maintained under specific pathogen-free conditions according to the guidelines set forth by the animal committee of the Tokyo Medical and Dental University (approval number A2019-207A).

### *In vitro* modification of BSA

The modification of BSA by aldehydes was performed by incubating BSA (1.0 mg/ml) with aldehydes in PBS for 24 h at 37 °C under atmospheric oxygen. After the incubation, aliquots were collected and dialyzed against PBS. For preparation of the biotin-labeled acrBSA, biotinylated BSA (1 mg/ml) was incubated with 10 mM acrolein in PBS at 37 °C under atmospheric oxygen. After 24 h, the aliquots were collected and dialyzed against PBS.

### Reaction of *N*^*α*^-acetylated amino acids with acrolein

The reaction mixture contained 10 mM *N*^*α*^-acetylated amino acids (Nα-acetyl-L-cysteine, Nα-acetyl-L-arginine, Nα-acetyl-L-histidine, or NAK) and 1 mM acrolein in PBS. After incubation for 24 h at 37 °C, the reaction mixtures were analyzed by reverse-phase HPLC using a Sunniest RP-Aqua column (5 μm, 4.6 × 250 mm, ChromaNik Technologies Inc) equilibrated in a solution of 5% methanol in 0.1% TFA at a flow rate of 1.0 ml/min. The elution profiles were monitored by absorbance at 200 to 600 nm and at 270 nm. The reaction provided one major product (peak a) and one minor product (peak b). Based on the LC-ESI-MS (ACQUITY TQD system, Waters) and NMR (Bruker Avance 400) spectrometry analyses, peaks a and b were identified as the *N*^*α*^-acetyl derivative of FDP-lysine and MP-lysine, respectively (17, 19).

### Isolation of splenic and PerC cells

SPLs were homogenized with a syringe, and the cells were passed through a 70-µm cell strainer using RPMI 1640 (Sigma) to obtain a single-cell suspension. All further steps were performed at 4 °C or on ice. Red blood cells were lysed using the red blood cell lysis buffer (eBioscience). For isolation of the PerC cells, peritoneal lavage was performed on sacrificed mice using 6 to 8 ml of RPMI 1640. Both the splenic and peritoneal cells were subsequently centrifuged at 300 g for 5 min and then resuspended in the medium.

### Isolation of B cells

The B220^+^ B cells were isolated using CD45R immunomagnetic beads. Briefly, the PerC cells (1 × 10^7^ cells) suspended in the magnetic-activated cell sorting (MACS) buffer (PBS/2 mM EDTA/0.5% BSA) were incubated with 10 μl of CD45R microbeads (Miltenyi Biotec) for 15 min at 4 °C. The cells were washed by adding 0.5 ml of the MACS buffer and centrifuged at 300*g* for 10 min. After aspirating the supernatant, the microbead-bound cells were resuspended in 0.5 ml of the buffer and the B cells were enriched using LS MACS columns (Miltenyi Biotech) according to manufacturers' instructions.

### *In vitro* incubation of cells with modified proteins

The cells (1 × 10^6^ cells/ml) prepared from mouse SPLs and PerC were dispensed onto 96-well plates (Corning Costar) in RPMI 1640 and incubated with the modified proteins (100 μg/ml) at 37 °C in 5% CO_2_ for 24 to 72 h. Stimulation of the PerC B cell subsets was performed under similar conditions. The supernatants of these cultures were collected and used in some experiments.

### Determination of secreted IgM

The levels of secreted total IgM were determined by ELISA. Goat anti-mouse IgM UNLB (SouthernBiotech) and goat anti-mouse IgM HRP (SouthernBiotech) were used as capture and detection Abs, respectively. A 100-μl aliquot of the capture Ab solution diluted with PBS was added to each well of a 96-well microtiter plate and incubated overnight at 4 °C. The plate was washed three times with PBS containing 0.5% Tween 20 (PBS/Tween), and each well was incubated with 200 μl of 4% blockace (Yukijirushi) in H_2_O for 1 h at room temperature (RT). The plate was then washed three times with PBS/Tween. A 100-μl aliquot of the supernatant samples diluted with PBS was added to each well and incubated for 2 h at RT. After discarding the supernatants and washing three times with PBS/Tween, 100 μl of a 5×10^3^ dilution of the detection Ab in PBS was added. After incubation for 1 h at RT, the supernatant was discarded, and the plate was washed three times with PBS/Tween. The enzyme-linked Ab bound to the well was revealed by adding 50 μl/well of 1-Step Ultra TMB solution (Thermo Fisher Scientific). The reaction was terminated by the addition of 2 N sulfuric acid (50 μl/well), and the absorbance at 450 nm was read using a 2030 Multilabel Reader ARVO X3 (PerkinElmer).

### Determinations of intracellular Ca^2+^ mobilization

Determinations of the intracellular Ca^2+^ mobilization in YC3.60-expressing cells were analyzed by confocal microscopy. For image acquisition of the peritoneal cells, a Nikon A1 laser-scanning confocal microscope with a 20× objective and NIS-Elements AR software were used, as described previously (16). We used dichroic mirrors (DM457/514) and two band-pass emission filters (482/35 for cyan fluorescent protein [CFP]; 540/30 for YFP). The YFP/CFP ratio was obtained by excitation at 458 nm. The acquired images were analyzed using the NIS-Elements software (Nikon).

### *In vitro* microscope

For image acquisition of the peritoneal cells, a Nikon A1 laser scanning confocal microscope with a 20× objective and NIS-Elements AR software were used, as described previously (16). We used dichroic mirrors (DM457/514) and two band-pass emission filters (482/35 for CFP; 540/30 for YFP). The YFP/CFP ratio was obtained by excitation at 458 nm. The acquired images were analyzed using the NIS-Elements software (Nikon).

### Flow cytometric sorting of PerC B cells

PerC cells were first incubated with anti-CD16/32 mAb (BioLegend) to block the Fc receptors on ice for 15 min before staining with the mAbs. The mAbs used for the lymphocyte staining were anti-mouse CD5-PE, anti-mouse CD19-APC, anti-mouse CD23-FITC, and anti-mouse CD138-PECy7 (BioLegend). Single-color controls and fluorescence minus one controls were included to ensure proper gating. The B cell populations were defined as total B cells (CD19^+^), B-2 cells (CD19^+^CD5^−^CD23^+^), B-1a cells (CD19^+^CD5^+^), and B-1b cells (CD19^+^CD5^−^CD23^−^). Each B cell subset was further stained using anti-CD138 mAb to detect the CD138^+^ plasma cells. The flow cytometric profiles were analyzed using FlowJo software (Becton, Dickinson and Company).

The binding of acrBSA to the PerC B cell subsets was also detected by flow cytometry. The PerC cells were first incubated with anti-CD16/32 Ab to block the Fc receptors on ice for 15 min and then incubated with biotin–acrBSA (200 μg/ml) for 15 min on ice. Immunostaining was performed at 4 °C for 20 min in 1% BSA–PBS using a mixture of anti-mouse CD5-PE, anti-mouse CD19-APC, and streptavidin PECy7 (BioLegend). All the analyses were performed using FlowJo.

### Preparation of acrolein-specific hybridoma and monoclonal Ab

The PerC cells from the BALB/c mice were fused with P3U1 murine myeloma cells and cultured in hypoxanthine/aminopterin/thymidine selection medium. The culture supernatants of the hybridoma were screened using an ELISA, using pairs of wells in microtiter plates on which were absorbed BSA or acrBSA as antigens (0.5 μg of protein per well). After incubation with 100 μl of the hybridoma supernatants, and with intervening washes with PBS/Tween, the wells were incubated with horseradish peroxidase goat anti-mouse IgM in PBS/Tween, followed by a substrate solution containing 100 μl/well of 1,2-phenylenediamine (0.5 mg/ml) in a 0.1 M citrate/phosphate buffer (pH 5.0) containing 0.03% hydrogen peroxide. Hybridoma cells, corresponding to the supernatants that were positive on the acrBSA and negative on the native BSA, were then cloned by limiting dilution. After repeated screenings, one clone RE-G25 showing the most distinctive recognition of acrBSA was obtained.

### Ab sequence analysis

Immunoglobulin variable region genes were cloned and sequenced after amplification by PCR. The total RNA was prepared from 5 × 10^6^ hybridoma cells by the Fast Gene RNA Basic Kit (NIPPON Genetics) according to manufacturer's protocol. The first-strand cDNA synthesis was performed with the PrimeScriptTM RT reagent Kit (TaKaRa) using the manufacturer's protocol. Subsequently, PCR was performed for amplification of VH and VL genes using antibody-specific primer sets, as previously described (33), and DNA sequencing of the products was carried out by Eurofins Genomics. The BLAST protocol was used to search the GenBank database to determine the homology with the V regions of other murine Abs that have been sequenced (34).

### Unbiased amplification and high-throughput sequencing of IgM–BCRs

The PerC B cells bound (ACR^+^) and unbound (ACR^−^) to acrBSA were sorted from PerC cells pooled from 11 individual mice, and the total RNAs were extracted using ISOGEN (Nippon Gene) following manufacturer's protocol. Unbiased amplification of IgM-BCRs was performed by adaptor-ligation PCR. The next-generation sequencing was entrusted to Repertoire Genesis Inc. Briefly, 100 ng of the total RNA was converted to cDNA. A specific primer BSL-18E containing polyT18 and a NotI sites was used for the cDNA synthesis. After the synthesis of dsDNA, a specific adaptor P10EA/P20EA was ligated to the blunted end of dsDNA and then the adaptor-ligated dsDNA was subjected to digestion with the NotI restriction enzyme. After clean up, PCR was performed with primer pairs specific for the constant region and the P20EA sequences. The second PCR was performed with a constant region-specific nested primer and a P20EA-specific primer. The primer sequences were previously reported. After amplification, index sequences were added using a Nextera XT Index Kit v2 SetA (Illumina). The indexed amplicons were mixed in an equimolar concentration, and the mixtures were subjected to next-generation sequencing on the Illumina MiSeq using paired-end read sequencing with a length of 2 × 300 bp. Theoretically, the total RNAs were extracted from approximately 10^5^ to 10^6^ B cells in each population, although a flow cytometric analysis was not routinely performed in this experiment. The suitable sequencing depth was predetermined as being approximately 100,000 reads per sample.

### Data analyses

Each sequence read was analyzed by the bioinformatics software created by Repertoire Genesis Inc. The identification of the V, D, and J regions was determined by identifying the sequence with the highest identity to reference sequence data sets available from the international ImMunoGeneTics information system database (http://www.imgt.org). The data processing, assignment, and data aggregation were automatically performed. The identical V, D, J, and deduced amino acid sequences of CDR3 were defined as a unique sequence read. A unique sequence read contained several variant sequences formed by somatic hypermutations, and thus, we considered the sequence reads sharing identical V, D, and J segments and identical amino acid sequences of CDR3 as an identical clonal lineage. The Repertoire Genesis software automatically counted the number of unique sequence reads and ranked them in the order of the copy number.

### Statistical analysis

Dunnett's and Tukey–Kramer's methods were used for multiple comparison tests. In some experiments, statistical analyses were performed using the unpaired Student's *t* test (GraphPad Prism; GraphPad Software). The paired Student's *t* test was used to compare the usage of the IGHV5 segment in two groups (ACR^+^ and ACR^−^ B cell populations). The *p* values <0.05 were considered significant.

## Data availability

All data are included within this article.

## Supporting information

This article contains supporting information.

## Conflict of interest

The authors declare that they have no conflicts of interest with the contents of this article.
