# Supplementary material for: Recognition of acrolein-specific epitopes by B cell receptors triggers an innate immune response
Source: J Biol Chem. 2021 Apr 9;296:100648. doi: 10.1016/j.jbc.2021.100648 (PMC8121969; doi:10.1016/j.jbc.2021.100648)
Supplement: Figures S1 to S6 [file mmc1.pdf]

Supporting information

Recognition of acrolein-specific epitopes by B cell receptors triggers an innate immune response

Ryunosuke Endo[1], Kazuki Uchiyama[1], Sei-Young Lim[1], Masanori Itakura[1], Takahiro Adachi[2], and Koji Uchida[1, 3, 4]

Table of Contents

Figure S1. Sorting of B cell subsets.

Figure S2. Representative flow-cytometry plots of the PerC B cells treated with biotin-BSA or biotin-acrBSA.

Figure S3. Representative flow-cytometry plots of PerC B-1a cells treated with biotin-BSA or biotin-acrBSA.

Figure S4. DNA and amino acid sequences of VH in IgM mAb RE-G25.

Figure S5. DNA and amino acid sequences of VL in IgM mAb RE-G25.

Figure S6. Sorting of acrolein-binding (ACR$^+$) and acrolein-non-binding (ACR$^-$) B cell populations from the PerC of BALB/c mice.

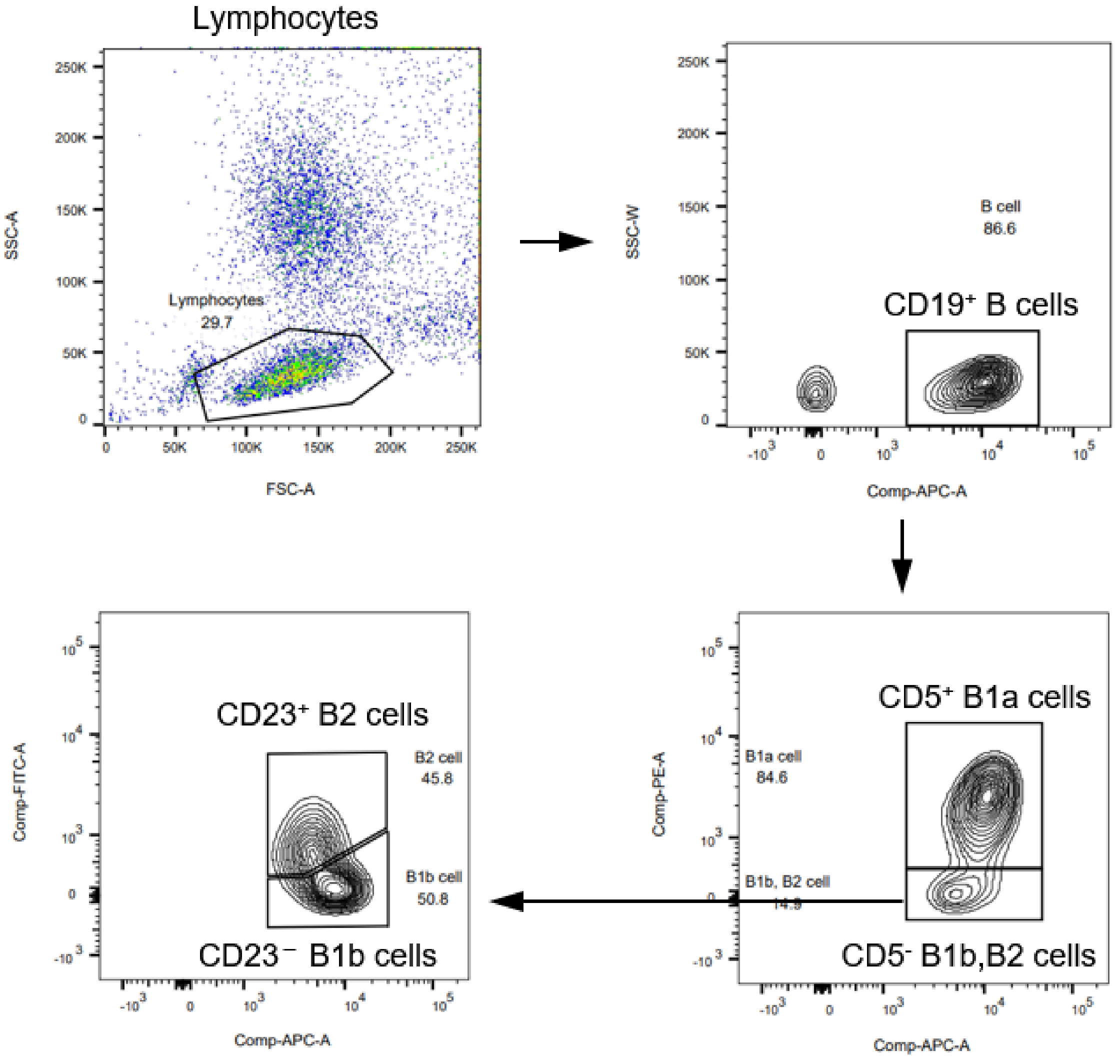

Figure S1. Sorting of B cell subsets.

B cell populations were defined as the total B cells ($CD19^{+}$), B-2 cells ($CD19^{+}CD5^{-}CD23^{+}$), B-1a cells ($CD19^{+}CD5^{+}$), and B-1b cells ($CD19^{+}CD5^{-}CD23^{-}$).

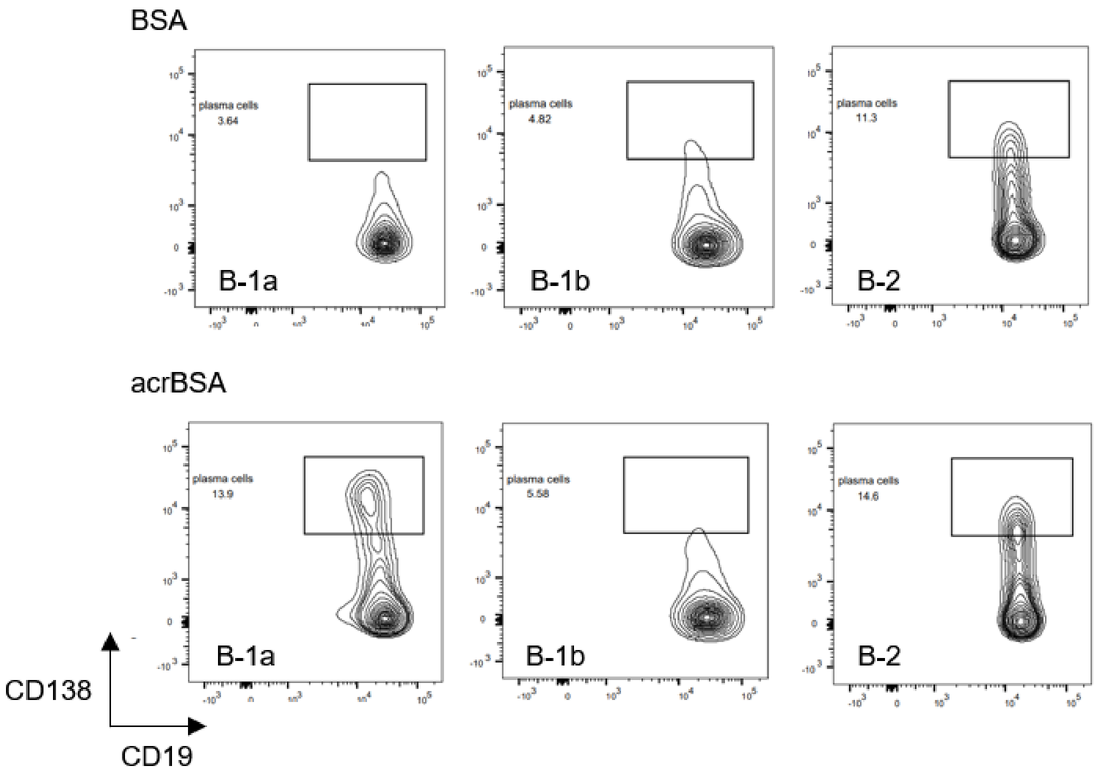

Figure S2. Representative flow-cytometry plots of the PerC B cells treated with biotin-BSA or biotin-acrBSA.

The PerC cells that had been treated with biotin-BSA (*upper three panels*) or biotin-acrBSA (*lower three panels*) for 72 h were sorted into the B cell subsets (B-1a, B-1b, and B-2 cells) and the changes in the number of plasma cells were measured. The gated B cell populations were stained for CD19 (anti-mouse CD19-APC) versus CD138 (anti-mouse CD138-FITC-PECy7).

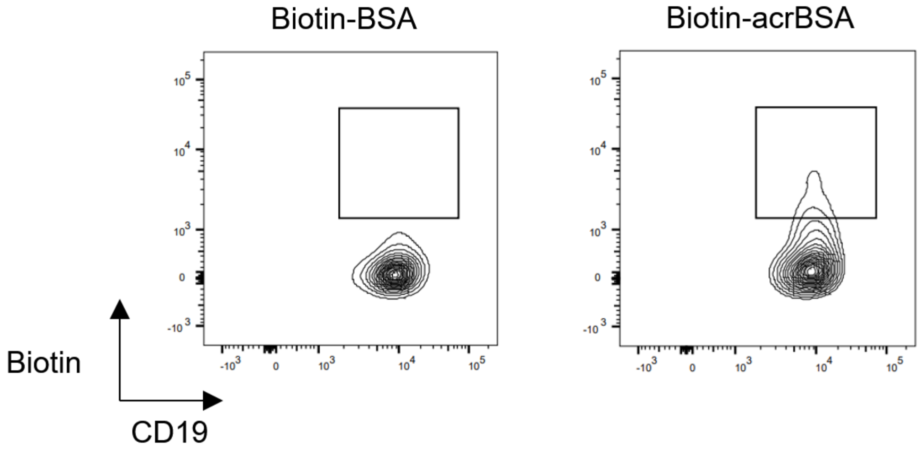

Figure S3. Representative flow-cytometry plots of PerC B-1a cells treated with biotin-BSA or biotin-acrBSA.

The PerC cells treated with biotin-BSA (*left*) or biotin-acrBSA (*right*) for 72 h were sorted into the B-1a cells and the changes in the number of antigen-binding cells were measured. The gated B-1a cell populations were stained for CD19 (anti-mouse CD19-APC) versus biotin (anti-mouse SA-PECy7).

VH: base sequence

GAGGTGCAGCTGGTGGAGTCTGGGGGAGACTTAGTGAAGCCTGGAGGGTC
CCTGAAACTCTCCTGTGCAGCCTCTGGATTCACTTTCAGTAGCTATGGCA
TGTCTTGGGTTCGCCAGACTCCAGACAAGAGGCTGGAGTGGGTCGCAACC
ATTAGTAGTGGTGGTAGTTACACCTACTATCCAGACAGTGTGAAGGGGCG
ATTCACCATCTCCAGAGACAATGCCAAGAACACCCTGTACCTGCAAATGA
GCAGTCTGAAGTCTGAGGACACAGCCATGTATTACTGTGCAAGACATGAT
TACGACTACTGGGGCCAAGGCACCACTCTCACAGTCTCCTCAG

VH: amino acid sequence

CDR1
EVQLVESGGDLVKPGGSLKLSCAASGFTFSSYGMSWVRQTPDKRLEWVAT
CDR2 CDR3
ISSGGSYTYYPDSVKGRFTISRDNAKNTLYLQMSSLKSEDTAMYYCARHD
YDYWGQGTTLTVSS

Figure S4. DNA and amino acid sequences of VH in IgM mAb RE-G25. The CDRs are highlighted.

VL: base sequence

GACATCCAGATGAACCAGTCTCCATCCAGTCTGTCTGCATCCCTTGGAGA

CACAATTACCATCACTTGCCATGCCAGTCAGAACATTAATGTTTGGTTAA

GCTGGTACCAGCAGAAACCAGGAAATATTCCTAAACTATTGATCTATAAG

GCTTCCAACTTGCACACAGGCGTCCCATCAAGGTTTAGTGGCAGTGGATC

TGGAACAGGTTTCACATTAACCATCAGCAGCCTGCAGCCTGAAGACATTG

CCACTTACTACTGTCAACAGGGTCAAAGTTATCCTCTCACGTTCGGTGCT

GGGACCAAGCTGGAAATAAAAC

VL: amino acid sequence

CDR1 CDR2

DIQMNQSPSSLSASLGDTITITCHASQNINVWLSWYQQKPGNIPKLLIYK

CDR3

ASNLHTGVPSRFSGSGSGTGFTLTISSLQPEDIATYYCQQGQSYPLTFGA

GTKLEIK

Figure S5. DNA and amino acid sequences of VL in IgM mAb RE-G25. The CDRs are highlighted.

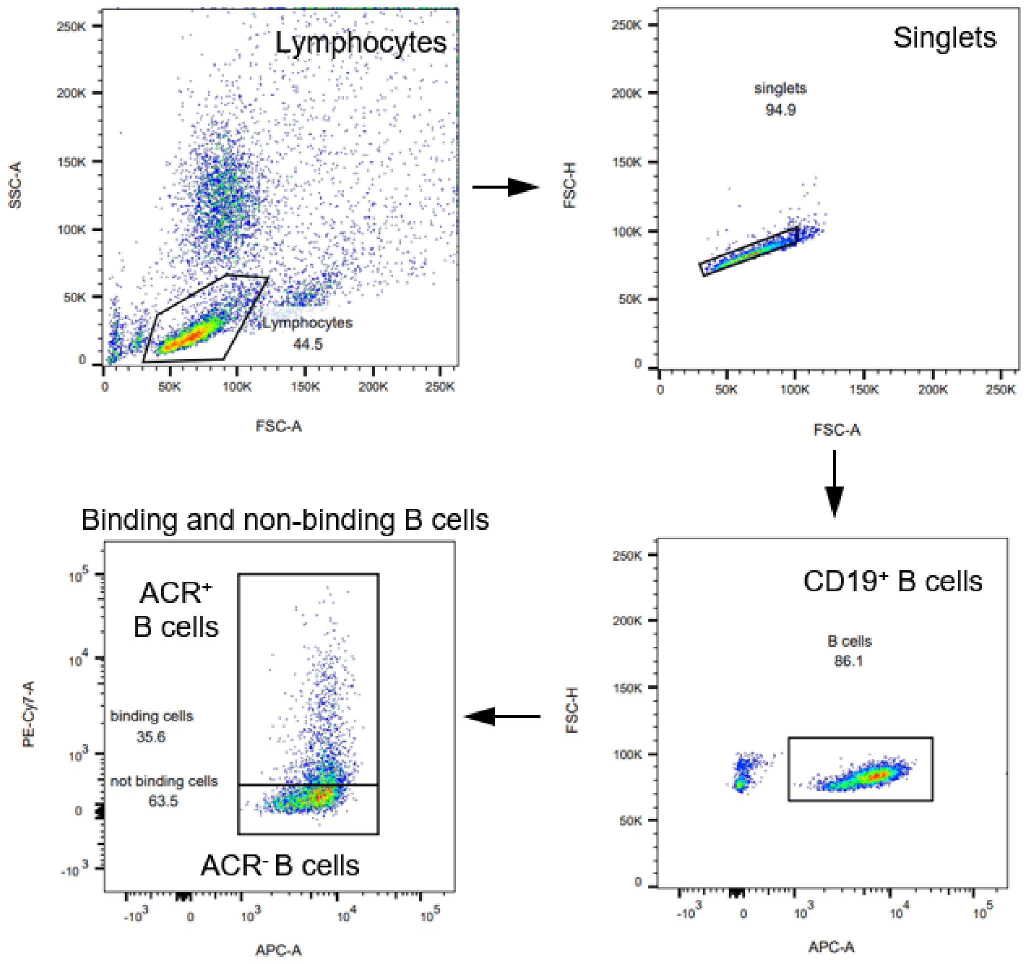

Figure S6. Sorting of acrolein-binding (ACR$^+$) and acrolein-non-binding (ACR$^-$) B cell populations from the PerC of BALB/c mice.
